# A systematic review on the clustering and co-occurrence of multiple risk behaviours

**DOI:** 10.1186/s12889-016-3373-6

**Published:** 2016-07-29

**Authors:** Nick Meader, Kristelle King, Thirimon Moe-Byrne, Kath Wright, Hilary Graham, Mark Petticrew, Chris Power, Martin White, Amanda J. Sowden

**Affiliations:** 1 Centre for Reviews and Dissemination, University of York, York, YO10 5DD UK; 2 Department of Health Sciences, University of York, York, UK; 3 Department of Social and Environmental Health Research, London School of Hygiene and Tropical Medicine, London, UK; 4 Population, Policy and Practice, UCL Institute of Child Health, London, UK; 5 UKCRC Centre for Diet and Activity Research (CEDAR), MRC Epidemiology Unit, University of Cambridge, Cambridge, UK

**Keywords:** Multiple risk behaviours, Systematic review, Clustering, Co-occurrence

## Abstract

**Background:**

Risk behaviours, such as smoking and physical inactivity account for up to two-thirds of all cardiovascular deaths, and are associated with substantial increased mortality in many conditions including cancer and diabetes. As risk behaviours are thought to co-occur in individuals we conducted a systematic review of studies addressing clustering or co-occurrence of risk behaviours and their predictors. As the main aim of the review was to inform public health policy in England we limited inclusion to studies conducted in the UK.

**Methods:**

Key databases were searched from 1990 to 2016. We included UK based cross-sectional and longitudinal studies that investigated risk behaviours such as smoking, physical inactivity, unhealthy diet. High heterogeneity precluded meta-analyses.

**Results:**

Thirty-seven studies were included in the review (32 cross-sectional and five longitudinal). Most studies investigated unhealthy diet, physical inactivity, alcohol misuse, and smoking. In general adult populations, there was relatively strong evidence of clustering between alcohol misuse and smoking; and unhealthy diet and smoking. For young adults, there was evidence of clustering between sexual risk behaviour and smoking, sexual risk behaviour and illicit drug use, and sexual risk behaviour and alcohol misuse.

The strongest associations with co-occurrence and clustering of multiple risk behaviours were occupation (up to 4-fold increased odds in lower SES groups) and education (up to 5-fold increased odds in those with no qualifications).

**Conclusions:**

Among general adult populations, alcohol misuse and smoking was the most commonly identified risk behaviour cluster. Among young adults, there was consistent evidence of clustering found between sexual risk behaviour and substance misuse. Socio-economic status was the strongest predictor of engaging in multiple risk behaviours.

This suggests the potential for interventions targeting multiple risk behaviours either sequentially or concurrently particularly where there is evidence of clustering. In addition, there is potential for intervening at the social or environmental level due to the strong association with socio-economic status.

**Electronic supplementary material:**

The online version of this article (doi:10.1186/s12889-016-3373-6) contains supplementary material, which is available to authorized users.

## Background

Behaviours such as lack of fruit and vegetable intake, physical inactivity, and smoking have been estimated to account for almost two-thirds of cardiovascular deaths in low-, middle- and high-income countries [1]. Similarly, engaging in four risk behaviours concurrently has been associated with a 3.35 fold increase in risk of mortality due to cancer [2]. Risk behaviours commonly co-occur. For example, 68 % of adults in England [3] 55 % in the Netherlands [4], 52 % in the USA [5] and 59 % in Brazil [6] were found to engage in two or more risk behaviours.

Developing an understanding of how risk behaviours co-occur (the prevalence of particular risk combinations) or cluster (where the risk behaviour combination is more frequent than predicted if they were independent) and which socio-demographic factors predict co-occurrence or clustering is of importance for public health policy and services globally [7, 8]. This information can usefully inform future prevention strategies.

Our review has two main aims. Firstly, to identify which risk behaviours cluster or co-occur. While there has been a systematic review [9] on the clustering of smoking, poor nutrition, alcohol and physical inactivity our review aimed to be more inclusive and to identify a broader range of risk behaviours. Secondly, to identify which socio-demographic factors are associated with multiple risk behaviours.

We used a broad search strategy and after mapping out this large and diverse literature focused on UK studies as the primary aim of the review was to inform public health policy in England.

## Methods

A systematic review was conducted and reported in accordance with current guidance [10, 11].

### Search strategy

We searched four electronic databases: MEDLINE, EMBASE, PsycINFO, and Science Citation Index from January 1990 to March 2016 (please see Additional file 1 for full search strategy). Electronic searches were supplemented by examination of the bibliographies of included studies and existing reviews.

### Inclusion criteria

#### Initial screening and mapping

Initially, studies eligible for inclusion were published after 1990, focused on adults (16 years of age and over) and examined the co-occurrence or clustering of two or more risk behaviours (e.g., alcohol misuse, smoking, physical inactivity, poor diet, illicit drug misuse, sexual risk behaviour, drink driving, lack of seat belt, motorcycle or bicycle helmet use, lack of sunscreen use, gambling, poor oral hygiene). We did not impose specific thresholds for risk behaviours as there is a lack of consensus in the literature for many behaviours. Therefore we included studies where thresholds for risk behaviours were reported and justified by authors. To minimise the risk of only identifying clustering and co-occurrence of behaviours which we had pre-determined the behaviours of potential interest were wide-ranging. No restrictions on study designs were applied.

Titles and abstracts were assessed by one author (KK) and checked by a second (NM). Any discrepancies were resolved by consensus and consultation with a third author (AJS) when necessary.

Of the 93,191 records identified 2258 were judged to be potentially relevant we used the abstracts to map the risk behaviours, predictors of risk behaviours, study design, and country for these studies. Because of the large number of records potentially meeting the inclusion criteria we then restricted inclusion to UK-based studies as the primary aim was to inform public health policy in the UK. UK and international studies did not appear to differ in the range of risk behaviours or predictors of risk clusters investigated justifying our decision.

### Data extraction

Data were extracted by one author (KK) and checked by a second (NM). Any discrepancies were resolved by consensus and consultation with a third reviewer (AJS) when necessary.

For all risk behaviours data on clustering (including cluster analysis, latent class analysis, prevalence odds ratios, regression analyses and any other measure of association) and co-occurrence (prevalence or percentages of co-occuring risk behaviours) were extracted. We also extracted data on the association between socio-demographic variables (e.g., age, gender, ethnicity, socio-economic status) and multiple risk behaviours (preferably specific risk cluster or behaviour combinations, but where these were not available we extracted data on risk indices such as 2, 3, 4 risk behaviours).

Data from multiple publications of the same study (or dataset) were extracted together and reported as a single study. All data were extracted and recorded under a primary reference (either the first published paper of the study or the paper including most relevant findings) with details of overlapping publications coded in the data extraction tool (EPPI Reviewer 4).

### Quality assessment

A recent systematic review of quality assessment tools [12] was consulted and the University of Wales College of Medicine [13] tool for the critical appraisal of observational studies was judged to be the most appropriate based on methodological relevance and ease of use.

We assessed appropriateness of study population, outcomes, study methods and sampling; response rate, potential for measurement bias, choice and use of statistical methods, applicability to research question and clarity of aims.

### Methods of analysis and synthesis

We conducted a narrative synthesis after investigation of the data suggested substantial conceptual (e.g. outcome definitions differed) and statistical heterogeneity. Studies were grouped based on population characteristics (e.g. students, older adults) and whether the aim was to examine co-occurrence or clustering.

We constructed forest plots to graphically explore patterns of similarity or difference across the studies’ findings using STATA 12 (co-occurrence) or Review Manager 5 (clustering) to inform the narrative synthesis (forest plots are available upon request).

## Results

**Fig. 1:**
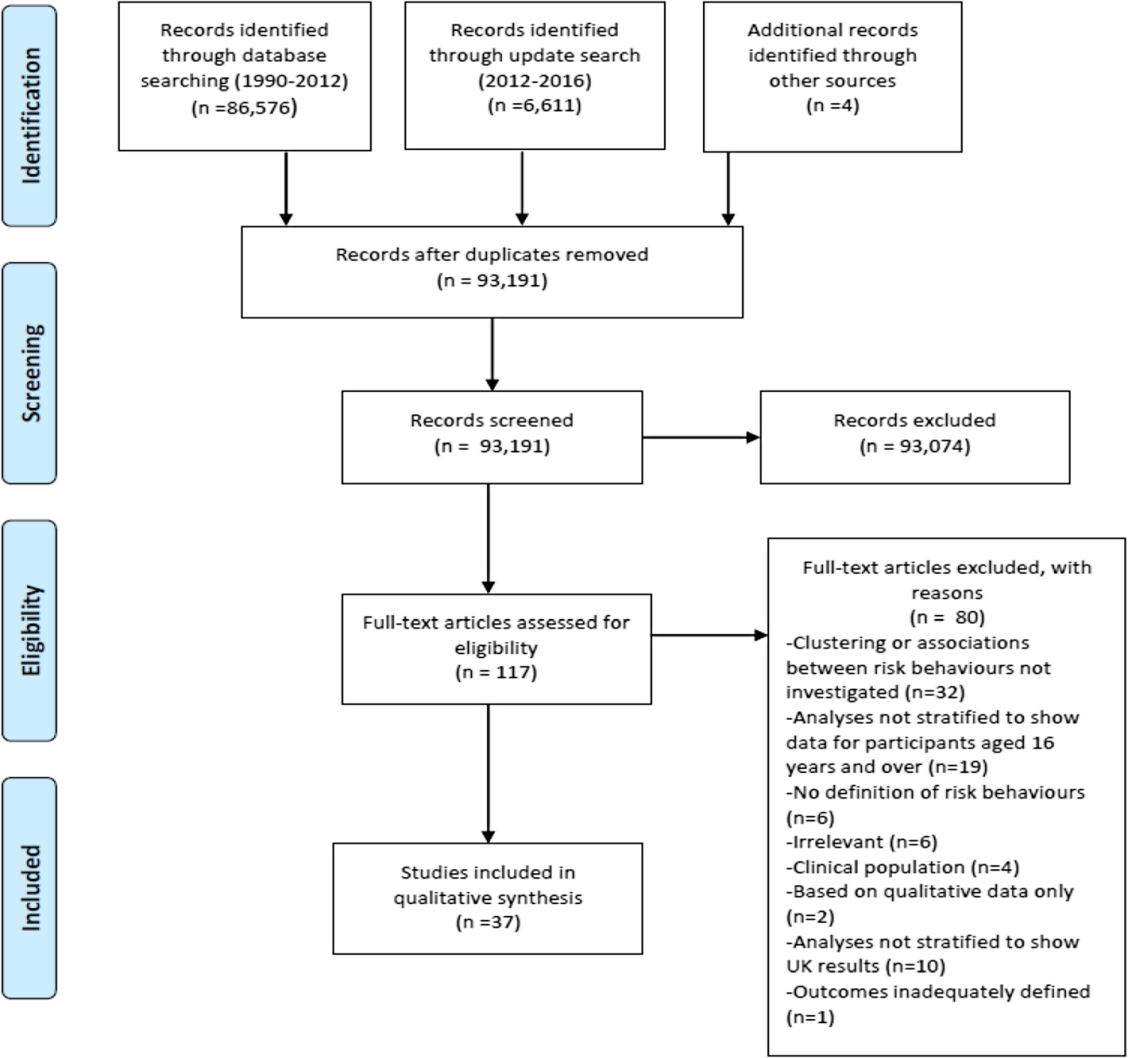
Study flow diagram

Searches identified 93, 191 records (see Fig. 1 for study flow diagram). Study characteristics are summarised in Additional file 2 and data for each combination of risk behaviours are provided in Table 1. After restricting to UK-based studies we conducted full text screening and 80 papers were excluded. The main reason for study exclusion was the lack of investigation of co-occurrence or of clustering between risk behaviours. Thirty-seven studies [3, 14–50] met the inclusion criteria and were included in the review.

**Table 1:** Summary table of co-occurrence, prevalence odds ratios and logistic regression analyses for combinations of two risk behaviours

Risk Behaviour combinations	Co-occurrence (prevalence range between studies)			Prevalence Odds Ratio (range between studies)			Odds Ratio from logistic regression analyses (range between studies)		
	Adults (16 years+)	Young adults (16–21 years)	Older adults (50 years +)	Adults (16 years+)	Young adults (16–21 years)	Older adults (50 years +)	Adults (16 years+)	Young adults (16–21 years)	Older adults (50 years +)
Low fruit and vegetables and low physical activity (2 studies)	47–54 %	-	-	1.19–1.67	-	-	-	-	-
	2 studies			2 studies					
	*N* = 18,066			*N* =18,066					
Alcohol misuse and smoking (12 studies)	9–14 %	13 %	3 %	1.81–2.89	0.90	1.32	1.55–2.44	-	-
	3 studies	1 study	1 study	3 studies	1 study	1 study	3 studies		
	*N* =26,045	*N* = 815	*N* = 11,214	*N* =26,045	*N* = 875	*N* = 11,214	*N* = 24,777		
Low fruit and vegetables and Alcohol misuse (2 studies)	13–26 %	-	-	1.09–1.63	-	-	-	-	-
	2 studies			2 studies					
	*N* = 18,066			*N* = 18,066					
Low Fruit and vegetables and Smoking (3 studies)	23–28 %	-	-	2.02–2.55	-	-	1.77	-	-
	3 studies			2 studies			1 study		
	*N* = 27,048			*N* = 18,066			*N* = 5553		
Physical activity and alcohol misuse (4 studies)	4–12 %	-	3 %	0.65–0.79	-	0.58	-	-	-
	3 studies		1 study	2 studies		1 study			
	*N* = 23,537		*N* = 11,214	*N* = 18,066		*N* = 11,214			
Physical activity and smoking (6 studies)	8–20 %	-	7 %	0.81–1.01	-	1.16 (2002)	-	-	-
						1.18 (2012)			
	5 studies		1 study	2 studies		1 study			
	*N* = 42,010		*N* = 11,214	*N* = 18,066		*N* = 11,214 (2012)			
Sexual risk and alcohol misuse (4 studies and 6 datasets)	-	-	-	-	-	-	1.81–2.77	1.38–3.22	-
							1 study/2 datasets	2 studies/3 datasets	
							*N* = 24,926	*N* = 3119	
Sexual risk and illicit drug use (3 studies and 4 datasets)	-	-	-	-	-	-	1.71–4.71	-	-
							3 studies/4 datasets		
							*N* = 4251		
Sexual risk and smoking (3 studies and 4 datasets)	-	-	-	-	-	-	1.71–2.11	-	-
							3 studies/4 datasets		
							*N* = 4251		

The main limitations of included studies were varying thresholds of risk behaviours. These limitations hinder comparison between studies and likely contribute to the observed heterogeneity in most of our data. For further details on quality assessment see summary table in Additional file 3.

### Risk behaviour co-occurrence and clustering

Most studies focused on general adult populations; with only a few studies investigating young adults, students and older adults.

The risk behaviour combinations and associated data are summarised in Table 1. Nine studies included two risk behaviours, 15 studies included three risk behaviours and 13 studies included more than three risk behaviours. The most common combinations of risk behaviours investigated were: alcohol and smoking, physical activity and smoking, and diet and smoking. The risk behaviours investigated appeared to depend on the target population.

Studies in adult populations most commonly investigated alcohol use and smoking, and physical inactivity and smoking. Most studies on young adults examined sexual risk behaviour combined with alcohol use, illicit drug use, or smoking. For all other subgroups there wasn’t sufficient data to conclude what behaviours was most commonly investigated.

Seventeen studies provided data on co-occurrence: adults were the focus in 11 studies, young adults in 2 studies and older adults in 1 study. Three of the seventeen studies focused on at risk populations but there were no studies of students (see online Appendices). For adult populations, the highest prevalence (range 47–54 %) for two risk behaviours was for low fruit and vegetable intake and low physical activity. This was based on two studies that included over 18,000 participants.

The combination of low fruit and vegetable intake and smoking was also high (range 23–38 %; three studies; over 27,000 participants). It was not possible to determine which behaviours were more likely to co-occur for young adults, older adults, students, and at risk populations due to a lack of studies.

Twenty-four studies provided data on clustering of risk behaviours, including adults (12 studies), students (three studies), young adults (4 studies, 5 datasets), older adults (one study), and at risk (i.e. more likely to be engaging in risk behaviours than the general population; 4 studies) populations. For most risk behaviours, although there were only a small number of studies their sample sizes were large (i.e. > 1000 participants).

For adult populations, the strongest evidence for clustering was found for alcohol misuse and smoking. The relatively large effect (Prevalence Odds Ratios (PORs) ranged from 1.81 to 2.89 and odds ratios (ORs) ranged from 1.55 to 2.44) indicates consistent associations between smoking and alcohol use and is based on over 20,000 participants.

For young adults, there was consistent evidence of a moderate to strong association (ORs ranged from 1.38 to 3.22) between sexual risk behaviour and alcohol use based on four studies (five datasets) from over 3000 participants. Similar findings (ORs ranged from 1.71 to 4.71) were observed for sexual risk behaviour and illicit drug use (three studies (four datasets), over 4000 participants) and sexual risk behaviour and smoking (ORs ranged from 1.71 to 2.11) three studies (four datasets), with over 4000 participants.

It was not possible to determine which behaviours cluster or co-occur in older adults, students, and at risk populations due to a lack of studies.

### Factors associated with multiple risk behaviours

Twelve studies (thirteen datasets) assessed factors associated with engaging in multiple risk behaviours. Six included adult populations [3, 14, 15, 21, 46, 50], three (four datasets) young adults [31, 44, 47], one students [36], one at risk populations [49] and one older adults [38].

Three used a cohort design [21, 31, 47] and nine a cross-sectional design [3, 14, 15, 36, 38, 44, 46, 49, 50]. Seven studies used an index of risk behaviours (e.g. any of 2, 3 or 4 risk behaviours) [3, 14, 15, 21, 38, 47, 50], three studies examined specific risk behaviour combinations [44, 46, 49], one study used cluster analysis [36], and one study latent class analysis [48].

Associations between gender, age, socio-economic status and ethnicity with co-occurrence or clustering of risk behaviours are summarised below.

#### Gender

Four studies included extensive adjustment for factors such as age, socio-economic status, and economic activity [3, 14, 15, 50] in adult populations and collectively they suggest that gender is a weak predictor of multiple risk behaviours. Three studies [3, 15, 50] found that males were more likely to engage in three or four risk behaviours. However another study [14] found that gender was not associated with engaging in two, three, or four risk behaviours (based on HSE 2008 data).

Two studies investigated the association between gender and risk behaviours in young adults. One study [48] used latent class analysis and did not find an association between gender and the ‘early smoking and heavy drinking’ category, but that females were more likely to belong to the ‘late smoking and heavy drinking’ category.

Another study [44] stratified observed/expected ratios by gender. Extent of clustering was similar for a number of risk behaviour combinations such as alcohol misuse and smoking (did not appear to cluster for males or females), drug misuse and alcohol misuse (less than expected for both males and females). However, there was evidence of clustering in males for the combination of drug misuse, smoking and alcohol misuse behaviours but not in females.

#### Age

Four studies examined age as a predictor of multiple risk behaviours in adult populations and carried out extensive adjustment for factors such as gender, socio-economic status, economic activity [3, 14, 15, 50]. Age as a predictor was inconsistent, for the two studies based in England [3, 14] 45–64 year olds were found to have lower odds of engaging in two, three or four risk behaviours compared with 16–24 year olds. But it was unclear whether there was a difference between 16–24 and 25–44 year age groups. A study of primary care practices in South Wales found that increased age was associated with fewer risk behaviours [50]. Scottish data [15] showed the opposite with older participants (25–34 years, 35–44 years, 45–54 years) reporting greater numbers of risk behaviours than younger participants (16–24 years).

#### Socio-economic status

The association between occupational group and multiple risk behaviours was assessed in four studies of adult populations [3, 14, 15, 50]. All analyses included extensive adjustment for factors such as age, gender and economic activity. The studies consistently showed that skilled manual, skilled non-manual, partially skilled and unskilled occupational groups were more likely to engage in two, three or four risk behaviours compared with professionals.

However, two cohort datasets reported in one paper [31] in young adults did not find an increased risk for those from manual occupational backgrounds engaging in sexual risk behaviour and substance use compared with people from non-manual backgrounds. A re-analysis [48] of one of the datasets (Twenty-07 Study) using latent class analysis found that young people from manual occupational backgrounds were more likely to belong to the ‘early smokers and heavy drinkers’ category (OR 1.89, 95 % CI 1.39 to 2.57) but not to the ‘late smokers and heavy drinkers’ category (OR 0.84, 95 % CI 0.43 to 1.65).

Another cohort study in young people did not find associations between low socio-economic status (as measured by receiving free lunches in high school) and engaging in two or more risk behaviours [47].

The education data were also consistent across general adult [14, 15] and older adult populations [38]. Those with no qualifications or intermediate qualifications were more likely to engage in multiple risk behaviours compared with those who attended higher education. For those with no qualifications there was a two-fold or greater increased odds of two, three, or four risk behaviours in most studies [14, 15, 38].

#### Ethnicity

Three studies examined the association between ethnicity and multiple risk behaviours. The three studies used different forms of analysis. One conducted regression analyses in a general population of adults which included adjustment for factors such as age, gender, socio-economic status [15]. One study conducted a cluster analysis in students [36]. Another study reported prevalence of risk behaviours for pregnant mothers separately by ethnicity [49].

All three studies suggested white participants were at greater risk of engaging in multiple risk behaviours than other ethnicities.

One study [15] found that people from black and minority ethnic (BME) groups were less likely to engage in two (RR 0.44; 95 % CI 0.23 to 0.83), three (RR 0.32; 95 % CI 0.16 to 0.65) or four (RR 0.16; 95 % CI 0.06 to 0.41) risk behaviours compared with white groups.

Another study [36] identified three clusters: unhealthy/high risk profile (e.g. low fruit and vegetable intake, lack of physical activity, high stress), moderately healthy/moderate risk profile (e.g. greater prevalence of smoking, low fruit and vegetable intake but also more physical activity), and healthy/low risk profile (e.g. low prevalence of smoking, higher fruit and vegetable intake and physical activity). White students were more likely to be in the moderate (91.6 %) or high risk (86.6 %) clusters. Asian or Asian British students were more likely to be in the low risk cluster (20.6 %), as were Black or Black British students (10.6 %).

A third study [49] found that smoking and binge-drinking was negligible in Pakistani, Indian, Bangladeshi, and Black pregnant women. The prevalence of concurrently engaging in these two risk behaviours was substantially higher in White (6.7 %) and ‘other ethnicities’ (mainly other White or Mixed Black and White ethnicity) (3.1 %).

## Discussion

### Principal findings on co-occurrence and clustering

We found consistent evidence of alcohol use and smoking clustering among adults independent of the alcohol use measure used. This is consistent with data from a number of countries including the Netherlands [4] and Hong Kong [51] where alcohol use and smoking had the strongest evidence of clustering of all the risk behaviours examined. The co-occurrence data showed a particularly high prevalence for low fruit and vegetable intake and low physical activity. This is consistent with data from other countries, including the United States [52], where physical inactivity and low fruit and vegetable intake were the most prevalent co-occurring behaviours.

Among young adults, there was strong and consistent evidence of sexual risk behaviour clustering with smoking, alcohol misuse, and illicit drug use. The association between sexual risk behaviour and smoking has received little research attention and is therefore particularly noteworthy.

Similar evidence of clustering has been found in Chinese college students [53], Korean adolescents [54], and adolescents from the United States [55]. This is surprising, given the often greater focus on associations between sexual risk behaviour and alcohol misuse or illicit drug use both in policy reports and reviews of intervention studies [56, 57].

### Principal findings on associations with multiple risk behaviours

There was a strong association between socio-economic status and the co-occurrence of risk behaviours in adults and older adults. For example, people with no educational qualifications had an approximate 2–6 fold increased odds of engaging in two, three or four risk behaviours compared with those who had been in higher education. This seems consistent with non-UK based studies which have also noted the importance of socio-economic status as a predictor of multiple risk behaviours [9, 58, 59].

Evidence concerning the impact of age as a predictor of multiple risk behaviours was inconsistent. Being in a younger age group was generally associated with greater risk [3, 14, 50] but another study did not find this association [15]. Similar findings were reported in a systematic review of international studies [9]. Gender appears to be weakly associated with multiple risk behaviours, with males at greater risk of engaging in 3 or 4 risk behaviours [3, 15, 50] although not replicated in another dataset [14]. This uncertainty also appears to be found in studies conducted outside of the UK. Some studies indicated a greater risk for males engaging in multiple risk behaviours (for example, in the United States [60]), whereas other studies did not find an association with gender (for example, a study of students in Germany [61]).

### Strengths and weaknesses

This is the first systematic review examining clustering and co-occurrence of a broad range of risk behaviours across adult populations. Previous reviews have tended to focus on pre-determined sets of behaviours such as physical inactivity, poor diet and smoking [9]. A particular strength of this systematic review was the use of an extensive search strategy (identifying over 90,000 records) that used a multi-faceted approach and investigating a range of potential strategies to minimise the possibility of missing relevant studies in the most resource efficient manner.

A potential limitation is that only UK studies were included in the review. However, this is also a strength as there is less uncertainty concerning the role of inter-country differences in explaining heterogeneity between studies. While including studies from outside of the UK may have influenced our conclusions (for example, for subgroups where we had limited data such as older adults), our findings appear consistent with the international literature concerning clustering and co-occurrence of risk behaviours [9].

A limitation of the current UK evidence base is that most studies examined the clustering of two risk behaviours, yet evidence suggests that many adults engage in three or more risk behaviours [14]. In addition, most studies used cross-sectional designs and there was insufficient data to be able to identify whether the data from cohort studies produced systematically different results.

Another limitation is the relatively small number of UK based studies that investigated factors associated with multiple risk behaviours such as age, gender, level of education. Although, most of the studies which did investigate predictors were large (sample size greater than 5000) nationally representative surveys with less risk of bias and our findings appear consistent with the wider literature. Another limitation is the lack of available data on the associations between socio-demographic variables and specific risk behaviour combinations. Most studies compare engaging in any risk behaviours with no risk behaviours. It is possible that the impact of age or gender may differ depending on which risk behaviour combination is examined and a generic measure of multiple risk behaviours (e.g. any two or three risk behaviours) cannot identify such patterns.

Another potential limitation was differences in definitions of risk behaviours used across the studies as this makes comparison between studies more difficult and contributes to the heterogeneity in our findings. Although more recent studies tended to use similar definitions which suggests this will be less important as the literature on multiple risk behaviours develops.

### Implications

Many of the risk behaviour clusters identified in our systematic review are well known (e.g. alcohol and smoking, unhealthy diet and smoking). However, the consistent clustering between smoking and sexual risk behaviour among young adults has received comparatively less research and policy attention. In addition, though SES is often acknowledged as a potential predictor of multiple risk behaviours the magnitude of impact suggests tackling these health inequalities should be a priority for public health policy and future interventions need to be accessible to socially disadvantaged groups.

### Unanswered questions and further research

Most studies examined clustering or co-occurrence between two risk behaviours. Few studies examined associations between three or more risk behaviours, despite evidence suggesting that many people engage in multiple risky behaviours. Priority should be given to the analysis of data from large datasets such as Biobank, Health Survey for England and Scottish Health Survey as well as in other national and international datasets.

Further research is also needed on factors associated with multiple risk behaviours particularly the impact of age and gender on specific risk behaviour combinations as the impact of age and gender may differ depending on the combination of risk behaviours targeted. In addition, further investigation is needed on factors associated with multiple risk behaviours in young adults, as we identified few UK based studies specifically examining this issue.

Most of the UK based behavioural research has focused on associations between diet, physical inactivity, alcohol misuse and smoking. The clustering of behaviours such as sexual risk taking, gambling and illicit drug misuse with other risk behaviours has been less extensively researched.

## Conclusions

Among general adult populations, alcohol misuse and smoking was the most commonly identified risk behaviour cluster. Among young adults, there was consistent evidence of clustering found between sexual risk behaviour and substance misuse. Socio-economic status was the strongest predictor of engaging in multiple risk behaviours.

This suggests the potential for interventions targeting multiple risk behaviours either sequentially or concurrently particularly where there is evidence of clustering. In addition, there is potential for intervening at the social or environmental level due to the strong association with socio-economic status.

## Additional files

Additional file 1: Search strategy. (DOCX 21 kb) Additional file 2: Study characteristics of included studies. (DOCX 20 kb) Additional file 3: Summary of quality assessments for each individual study. (DOCX 26 kb)
