# Supplementary material for: A systematic review on the clustering and co-occurrence of multiple risk behaviours
Source: BMC Public Health. 2016 Jul 29;16:657. doi: 10.1186/s12889-016-3373-6 (PMC4966774; doi:10.1186/s12889-016-3373-6)
Supplement: Additional file 1: — Search strategy. (DOCX 21 kb) [file 12889_2016_3373_MOESM1_ESM.docx]

**Supplementary files 2: Search strategy**

**Ovid MEDLINE(R) In-Process & Other Non-Indexed Citations and Ovid MEDLINE(R) <1946 to Present>**

**Search date: 23^rd^ December 2012**

--------------------------------------------------------------------------------

1 (health$ adj2 (diet$ or eating or food or foods)).ti,ab. (9220)

2 (unhealth$ adj2 (diet$ or eating or food or foods)).ti,ab. (987)

3 fruit/ or vegetables/ (29321)

4 (fruit$ adj2 (eat or eats or eating or intake or consum$ or increas$ or portion$ or serving$ or frequenc$ or number$ or preference$ or choice$)).ti,ab. (5091)

5 (vegetable$ adj2 (eat or eats or eating or intake or consum$ or increas$ or portion$ or serving$ or frequenc$ or number$ or preference$ or choice$)).ti,ab. (4625)

6 "5 a day".mp. (126)

7 "five a day".ti,ab. (26)

8 (junk food or fast food).ti,ab. (1257)

9 ((decreas$ or reduc$ or discourag$ or limit$ or lessen or eat$ less) adj2 (salt or fat)).ti,ab. (8929)

10 (food adj (choice$ or frequenc$ or select$)).ti,ab. (7988)

11 Feeding Behavior/ (33125)

12 food habits/ or food preferences/ (24048)

13 nutrition therapy/ or exp diet therapy/ (37188)

14 obesity/ or overweight/ (105809)

15 or/1-14 (229653)

16 (comment or editorial or letter).pt. (1144084)

17 15 not 16 (219188)

18 limit 17 to yr="1990 -Current" (156354)

19 (physical$ adj3 (fit$ or train$ or activ$ or inactiv$ or endur$)).ti,ab. (57920)

20 (exercis$ adj3 (fit$ or train$ or activ$ or endur$)).ti,ab. (19437)

21 ((promot$ or uptak$ or encourag$ or increas$ or start$ or adher$) adj3 (exercis$ or gym$ or sport$ or fitness)).ti,ab. (19479)

22 ((decreas$ or reduc$ or discourag$) adj3 (sedentary or deskbound)).ti,ab. (364)

23 (sedentary behaviour$ or sedentary behavior$ or sedentary lifestyle$ or sedentariness).ti,ab. (2714)

24 sedentary lifestyle/ (939)

25 ((watch$ or view$) adj2 (tv or television)).ti,ab. (2304)

26 (sport$ or walk$ or running or jogging or bicycling or biking or swimming).ti,ab. (135633)

27 (active adj (travel$ or transport$ or commut$)).ti,ab. (6532)

28 physical fitness/ (19149)

29 exp Recreation/ or leisure activities/ (113329)

30 exp Exercise Therapy/ or Exercise/ (78276)

31 running/ or jogging/ or swimming/ or walking/ (36153)

32 or/19-31 (326294)

33 (letter or editorial or comment).pt. (1144084)

34 32 not 33 (314737)

35 limit 34 to yr="1990 -Current" (246568)

36 exp smoking/ (106047)

37 (smoking or antismoking or anti-smoking).ti,ab. (124050)

38 (smoker or smokers).ti,ab. (49322)

39 tobacco/ or tobacco.ti,ab. (63819)

40 36 or 37 or 38 or 39 (211282)

41 (letter or editorial or comment).pt. (1144084)

42 40 not 41 (201949)

43 limit 42 to yr="1990 -Current" (159226)

44 exp Alcohol Drinking/ (44202)

45 exp Alcoholic Intoxication/ (10157)

46 exp Alcoholic Beverages/ (11911)

47 exp Drinking Behavior/ (49358)

48 (beer or wine$ or cider or alcopop$ or spirit or spirits).ti,ab. (19044)

49 alcohol$.ti,ab. (200605)

50 (drink$ adj2 (binge or excessive or harm$ or heavy or misus$ or abus$ or consum$)).ti,ab. (9119)

51 (intoxicat$ or inebriat$ or drunk$).ti,ab. (36346)

52 44 or 45 or 46 or 47 or 48 or 49 or 50 or 51 (264632)

53 (comment or editorial or letter).pt. (1144084)

54 52 not 53 (257016)

55 limit 54 to yr="1990 -Current" (177578)

56 Unsafe Sex/ (1936)

57 multiple sexual partner$.ti,ab. (697)

58 multiple casual partner$.ti,ab. (10)

59 one time sex$ encounter$.ti,ab. (3)

60 one-time sex$ encounter$.ti,ab. (3)

61 (sex$ adj2 holiday$).ti,ab. (8)

62 casual sex$.ti,ab. (451)

63 casual partner$.ti,ab. (600)

64 non-regular sex$ partner$.ti,ab. (22)

65 non regular sex$ partner$.ti,ab. (22)

66 (unprotected adj2 intercourse).ti,ab. (1746)

67 (unprotected adj2 sex$).ti,ab. (2344)

68 (condomless adj (sex or intercourse)).ti,ab. (7)

69 (condom free adj (sex or intercourse)).ti,ab. (2)

70 (RUAI or UAI).ti,ab. (236)

71 (barebacking or bareback sex$ or bugchas$ or bug chas$).ti,ab. (59)

72 anal intercourse.ti,ab. (1373)

73 anal sex.ti,ab. (960)

74 or/56-73 (7225)

75 sexual behavior/ (36589)

76 risk taking/ (15617)

77 75 and 76 (4504)

78 (risk$ sex$ behavio$ or unsafe sex$).ti,ab. (2666)

79 74 or 77 or 78 (11703)

80 (letter or comment or editorial).pt. (1144084)

81 79 not 80 (11384)

82 limit 81 to yr="1990 -Current" (11011)

83 substance-related disorders/ or inhalant abuse/ or marijuana abuse/ or substance abuse, intravenous/ (82985)

84 Drug Users/ (605)

85 ((drug$ or substance$) adj2 (use$ or using or abuse$ or abusing or misuse$ or misusing)).ti,ab. (119941)

86 ((heroin or opiate$ or cocaine or crack) adj2 (use$ or using or abuse$ or abusing or misuse$ or misusing)).ti,ab. (11375)

87 ((cannabis or marijuana) adj2 (use$ or using or abuse$ or abusing or misuse$ or misusing)).ti,ab. (5635)

88 ((benzodiazepine$ or amphetamine$ or methamphetamine$ or MDMA or ecstasy) adj2 (use$ or using or abuse$ or abusing or misuse$ or misuising)).ti,ab. (5612)

89 (solvent$ adj2 (use$ or using or abuse$ or abusing or misuse$ or misusing)).ti,ab. (5485)

90 street drug$.ti,ab. (397)

91 (prescri$ drug$ adj2 (use$ or using or abuse$ or abusing or misuse$ or misusing)).ti,ab. (1099)

92 polydrug use$.ti,ab. (531)

93 inject$ drug use$.ti,ab. (6780)

94 (needle$ adj3 (share$ or sharing)).ti,ab. (1190)

95 (syringe$ adj3 (share$ or sharing)).ti,ab. (431)

96 or/83-95 (178426)

97 (letter or editorial or comment).pt. (1144084)

98 96 not 97 (170668)

99 limit 98 to yr="1990 -Current" (132081)

100 sunbathing/ (178)

101 sunscreening agents/ (3374)

102 (sunbath$ or sunscreen$ or sunburn$ or suntan$ or sunbed$).ti,ab. (4849)

103 (sun bath$ or sun screen$ or sun burn$ or sun tan$ or sun bed$).ti,ab. (245)

104 sun protect$.ti,ab. (1665)

105 (tanning adj (bed$ or salon$ or studio$)).ti,ab. (166)

106 100 or 101 or 102 or 103 or 104 or 105 (7059)

107 (letter or comment or editorial).pt. (1144084)

108 106 not 107 (6590)

109 limit 108 to yr="1990 -Current" (5579)

110 dental care/ or dental caries/ (46076)

111 oral hygiene/ or toothbrushing/ (13570)

112 (dental care or dental health or dental hygiene).ti,ab. (15079)

113 (oral care or oral health or oral hygiene).ti,ab. (18102)

114 (gingival care or gingival health or gingival hygiene).ti,ab. (702)

115 ((unsupervised or irregular$ or regular$ or seldom or lack or never or infrequent$ or frequen$ or insufficient$) adj2 (toothbrushing or flossing)).ti,ab. (320)

116 ((irregular$ or regular$ or seldom or lack or never or infrequent$ or frequen$) adj3 (dental or dentist$)).ti,ab. (2363)

117 ((irregular$ or regular$ or seldom or lack or never or infrequent$ or frequen$) adj3 dental visit$).ti,ab. (259)

118 ((irregular$ or regular$ or seldom or lack or never or infrequent$ or frequen$) adj3 dental attendance).ti,ab. (79)

119 (clean$ teeth adj2 (irregular$ or regular$ or infrequent$ or frequen$ or never or seldom)).ti,ab. (3)

120 (brush$ teeth adj2 (irregular$ or regular$ or infrequent$ or frequen$ or never or seldom)).ti,ab. (9)

121 ((sweet$ drink$ or fizzy drink$ or sugary snack$ or sweets or confectionery) adj6 (tooth or teeth or dental or oral or caries or decay)).ti,ab. (145)

122 or/110-121 (74333)

123 (letter or editorial or comment).pt. (1144084)

124 122 not 123 (71117)

125 limit 124 to yr="1990 -Current" (37061)

126 patient compliance/ (41426)

127 treatment refusal/ (9855)

128 126 and 127 (776)

129 (non-adherence adj2 (patient$ or medication$ or screen$ or treatment or therapy or immunisation or immunization or regimen$ or drug$)).ti,ab. (510)

130 (nonadherence adj2 (patient$ or medication$ or screen$ or treatment or therapy or immunisation or immunization or regimen$ or drug$)).ti,ab. (752)

131 (low adherence adj2 (patient$ or medication$ or screen$ or treatment or therapy or immunisation or immunization or regimen$ or drug$)).ti,ab. (60)

132 (poor adherence adj2 (patient$ or medication$ or screen$ or treatment or therapy or immunisation or immunization or regimen$ or drug$)).ti,ab. (325)

133 ((loss or lack or failure or barrier$ or impediment$ or selective or minimal) adj2 adherence).ti,ab. (1012)

134 (non-compliance adj2 (patient$ or medication$ or screen$ or treatment or therapy or immunisation or immunization or regimen$ or drug$)).ti,ab. (674)

135 (noncompliance adj2 (patient$ or medication$ or screen$ or treatment or therapy or immunisation or immunization or regimen$ or drug$)).ti,ab. (1080)

136 (low compliance adj2 (patient$ or medication$ or screen$ or treatment or therapy or immunisation or immunization or regimen$ or drug$)).ti,ab. (86)

137 (poor compliance adj2 (patient$ or medication$ or screen$ or treatment or therapy or immunisation or immunization or regimen$ or drug$)).ti,ab. (397)

138 ((loss or lack or failure or barrier$ or impediment$ or selective or minimal) adj2 compliance).ti,ab. (1002)

139 treatment refusal/ (9855)

140 mass screening/ (72173)

141 139 and 140 (127)

142 (non-attend$ adj3 screen$).ti,ab. (49)

143 (nonattend$ adj3 screen$).ti,ab. (17)

144 (non-attend$ adj3 appoint$).ti,ab. (36)

145 (nonattend$ adj3 appoint$).ti,ab. (17)

146 (non-attend$ adj3 (check-up$ or checkup$)).ti,ab. (3)

147 (nonattend$ adj3 (check-up$ or checkup$)).ti,ab. (0)

148 (non-attend$ adj3 (mammograph$ or smear test$ or PAP test$ or breast exam$ or CBE)).ti,ab. (11)

149 (nonattend$ adj3 (mammograph$ or smear test$ or PAP test$ or breast exam$ or CBE)).ti,ab. (5)

150 128 or 129 or 130 or 131 or 132 or 133 or 134 or 135 or 136 or 137 or 138 or 141 or 142 or 143 or 144 or 145 or 146 or 147 or 148 or 149 (6575)

151 (letter or editorial or comment).pt. (1144084)

152 150 not 151 (6431)

153 limit 152 to yr="1990 -Current" (5868)

154 seat belts/ (3143)

155 (seatbelt$ or seat belt$).ti,ab. (2657)

156 seat restraint$.ti,ab. (40)

157 passenger$ restraint$.ti,ab. (52)

158 driver$ restraint$.ti,ab. (11)

159 ((unbelted or unrestrained) adj2 (driver$ or passenger$)).ti,ab. (108)

160 154 or 155 or 156 or 157 or 158 or 159 (4256)

161 head protective devices/ (2129)

162 (cycle helmet$ or bike helmet$ or bicycle helmet$).ti,ab. (480)

163 161 or 162 (2218)

164 protective devices/ (5460)

165 fires/ or smoke/ (10879)

166 164 and 165 (154)

167 (smoke adj (alarm$ or sensor$)).ti,ab. (133)

168 (fire adj (alarm$ or sensor$)).ti,ab. (47)

169 166 or 167 or 168 (285)

170 automobile driving/ (11762)

171 alcoholic intoxication/ or alcohol drinking/ (52148)

172 170 and 171 (2255)

173 (drink$ adj2 (drive$ or driving)).ti,ab. (1161)

174 alcohol impaired driv$.ti,ab. (188)

175 172 or 173 or 174 (2850)

176 160 or 163 or 169 or 175 (9267)

177 (editorial or letter or comment).pt. (1144084)

178 176 not 177 (8500)

179 limit 178 to yr="1990 -Current" (6146)

180 Gambling/ (2666)

181 (gambling or gambler).mp. or gamblers.ti,ab. [mp=title, abstract, original title, name of substance word, subject heading word, protocol supplementary concept, rare disease supplementary concept, unique identifier] (3571)

182 180 or 181 (3571)

183 (letter or editorial or comment).pt. (1144084)

184 182 not 183 (3361)

185 limit 184 to yr="1990 -Current" (2987)

186 (18 and 35) or (18 and 43) or (18 and 55) or (18 and 82) or (18 and 99) or (18 and 109) or (18 and 125) or (18 and 153) or (18 and 179) or (18 and 185) (33147)

187 (35 and 18) or (35 and 43) or (35 and 55) or (35 and 82) or (35 and 99) or (35 and 109) or (35 and 125) or (35 and 153) or (35 and 179) or (35 and 185) (35046)

188 (43 and 18) or (43 and 35) or (43 and 55) or (43 and 82) or (43 and 99) or (43 and 109) or (43 and 125) or (43 and 153) or (43 and 179) or (43 and 185) (44075)

189 (55 and 18) or (55 and 35) or (55 and 43) or (55 and 82) or (55 and 99) or (55 and 109) or (55 and 125) or (55 and 153) or (55 and 179) or (55 and 185) (53767)

190 (82 and 18) or (82 and 35) or (82 and 43) or (82 and 55) or (82 and 99) or (82 and 109) or (82 and 125) or (82 and 153) or (82 and 179) or (82 and 185) (4600)

191 (99 and 18) or (99 and 35) or (99 and 43) or (99 and 55) or (99 and 82) or (99 and 109) or (99 and 125) or (99 and 153) or (99 and 179) or (99 and 185) (32110)

192 (109 and 18) or (109 and 35) or (109 and 43) or (109 and 55) or (109 and 82) or (109 and 99) or (109 and 125) or (109 and 153) or (109 and 179) or (109 and 185) (659)

193 (125 and 18) or (125 and 35) or (125 and 43) or (125 and 55) or (125 and 82) or (125 and 99) or (125 and 109) or (125 and 153) or (125 and 179) or (125 and 185) (3534)

194 (153 and 18) or (153 and 35) or (153 and 43) or (153 and 55) or (153 and 82) or (153 and 99) or (153 and 109) or (153 and 125) or (153 and 179) or (153 and 185) (1035)

195 (179 and 18) or (179 and 35) or (179 and 43) or (179 and 55) or (179 and 82) or (179 and 99) or (179 and 109) or (179 and 125) or (179 and 153) or (179 and 185) (3183)

196 (185 and 18) or (185 and 35) or (185 and 43) or (185 and 55) or (185 and 82) or (185 and 99) or (185 and 109) or (185 and 125) or (185 and 153) or (185 and 179) (1023)

197 186 or 187 or 188 or 189 or 190 or 191 or 192 or 193 or 194 or 195 or 196 (96292)

198 exp animals/ not humans.sh. (3644792)

199 197 not 198 (92397)

200 cohort studies/ or longitudinal studies/ or retrospective studies/ or prospective studies/ or cross sectional studies/ or follow-up studies/ or epidemiologic studies/ (1245985)

201 (cohort adj (study or studies)).tw. (59543)

202 cohort analy$.tw. (2709)

203 ("follow up" adj (study or studies)).tw. (33042)

204 (observational adj (study or studies)).tw. (30873)

205 (descriptive adj (study or studies)).tw. (12030)

206 (epidemiologic$ adj (study or studies)).tw. (51931)

207 longitudinal.tw. (111494)

208 retrospective.tw. (213679)

209 prospective.tw. (286276)

210 cross sectional.tw. (123369)

211 health surveys/ (38746)

212 or/200-211 (1575862)

213 199 and 212 (33584)
