# Supplementary material for: A systematic review on the clustering and co-occurrence of multiple risk behaviours
Source: BMC Public Health. 2016 Jul 29;16:657. doi: 10.1186/s12889-016-3373-6 (PMC4966774; doi:10.1186/s12889-016-3373-6)
Supplement: Additional file 2: — Study characteristics of included studies. (DOCX 20 kb) [file 12889_2016_3373_MOESM2_ESM.docx]

**Supplementary file 1: Study characteristics of the included studies**

| **Author/s (date of publication), location** | **Study population** | **Study Design** | | **Date of data collection (and name of survey if available)** | | **Risk behaviours investigated** | **Predictors of multiple risk behaviours investigated** | **Clustering or**  **co-occurrence investigated** |
| --- | --- | --- | --- | --- | --- | --- | --- | --- |
| **General adult population (study did not restrict inclusion criteria to specific at risk or age groups)** | | | | | | | | |
| Aicken *et al.* (2011)  England, Scotland, Wales, | General population (N=24,296) | | Cross-sectional | | NATSAL 1: 1990-1991  NATSAL 2: 2000-2001 | Alcohol misuse, sexual risk behaviour | - | Clustering |
| Buck & Frosini (2012)  England | General population (N=14,912) | | Cross-sectional | | HSE 2003 and 2008 | Alcohol misuse, smoking, low levels of fruit and vegetable intake, low level of physical activity | Gender, age, occupational group, education, employment status | - |
| Griffiths *et al.* (2010, 2011)  England, Scotland, Wales | General population (N=9,003) | | Cross-sectional | | British Gambling Prevalence Survey 2007 | Alcohol misuse, gambling, smoking | - | Co-occurrence and clustering |
| Lawder *et al.* (2010)  Scotland | General population (N=6,574) | | Cross-sectional | | Scottish Health Survey 2003 | Alcohol misuse, low level of physical activity/exercise, low level of fruit and vegetable intake, smoking | Gender, age, occupational group, deprivation of geographical area, education, employment status, ethnicity, marital status | Co-occurrence and clustering |
| Liao *et al.* (1995)  England | General population (45-year old women) (N=106) | | Cross-sectional | | 1991-1992 | Alcohol misuse, low level of physical activity/exercise, smoking | - | Clustering |
| Plant *et al.* (2002)  England, Scotland, and Wales | General population (N=2,027) | | Cross-sectional | | 2000 | Alcohol misuse, drug misuse, smoking | - | Clustering |
| Poortinga (2007)  England | General population (N=11,492) | | Cross-sectional | | HSE 2003 | Alcohol misuse, low level of physical activity/exercise, low level of fruit and vegetable intake, smoking | Gender, age, occupational group, education, employment status | Co-occurrence and clustering |
| Randell *et al.* (2015)  Wales | General population  (N=2,066) | | Cross-sectional | | Baseline data for Pre-Empt trial 2010 | Alcohol misuse, smoking, low level of physical activity, and unhealthy diet | Age, gender, marital status, occupation | Co-occurrence |
| Sabia *et al.* (2009)  England | General population (N=5,123) | | Prospective Cohort | | Whitehall II study  1985-1988 | Alcohol misuse, low level of physical activity/exercise, low level of fruit and vegetable intake, smoking | Age, gender, employment grade | - |
| Singh *et al.* (2013) | General population (N=11,380) | | Cross-sectional | | Adult Dental Health Survey 2009 | Smoking, poor oral health (tooth brushing less than twice a day, dental attendance only in trouble/never, high sugar consumption) | Education | Co-occurrence and clustering |
| Tang *et al.* (1997) England | General population (N=8,109) | | Cross-sectional | | OXCHECK  1989-1993 | Alcohol misuse, other dietary intake, smoking | - | Clustering |
| Thompson *et al.* (1999)  England | General population (N=5,553) | | Cross-sectional | | Health and Lifestyle Survey 1993 | Low level of fruit and vegetable intake, smoking | - | Clustering |
| Thornton *et al.* (1994)  England, Scotland, and Wales | General population (N=9,003) | | Cross-sectional | | Health and Lifestyle Survey 1987 | Alcohol misuse, low level of physical activity/exercise, low level of fruit and vegetable intake, other dietary intake, smoking | - | Co-occurrence |
| Uitenbroek (1993)  Scotland | General population (N=5,471) | | Cross-sectional | | 1991 | Alcohol misuse, low level of physical activity/exercise, other dietary intake, sexual risk behaviour, lack of seat belt use, smoking | - | Co-occurrence and Clustering |
| Uitenbroek (1994)  England, Scotland | General population (N=7,717) | | Cross-sectional | | 1990 | Alcohol misuse, low level of physical activity/exercise, sexual risk behaviour, lack of seat belt use, drink driving, smoking | - | Co-occurrence |
| Underwood *et al.* (2007)  UK | General population (Vocational dental practitioners) (N=767) | | Cross-sectional | | 2005 | Alcohol misuse, drug misuse, smoking | - | Co-occurrence and Clustering |
| Wadsworth *et al.* (2004)  Wales | General population (N=7,979) | | Cross-sectional | | 2001 | Alcohol misuse, drug misuse, smoking | - | Co-occurrence and Clustering |
| Woodward *et al.* (1994)  Scotland | General population (N=9,491) | | Cross-sectional | | Scottish Heart Health Study  1984-1986 | Alcohol misuse, low level of physical activity/exercise, other dietary intake, smoking | - | Co-occurrence |
| **Young adults (study inclusion criteria restricted to ages 16-21 years)** | | | | | | | | |
| Egginton *et al.* (2002)  England | Young adults (N=815) | Cross-sectional | | | Northern Regional Longitudinal Study  2000 | Alcohol misuse, drug misuse, smoking | - | Co-occurrence |
| Hale *et al.* (2013)  England | Young adults (N=9,467) | Prospective Cohort | | | Longitudinal Study of Young People in England 2010 | Sexual risk behaviour, alcohol use, drug misuse | Socio-economic status | - |
| Jackson *et al.* (2012)  Scotland  Green *et al.* (2013) | Young adults (Earlier cohort: N=908; later cohort: N=1258) | Prospective Cohort | | | Twenty-07 Study: Health in the community (Earlier cohort): 1990  (Jackson et al. 2012; Green et al. 2013)  11-16/16+ Study: Young People’s Health  (Later cohort): 2003  (Jackson et al. 2012) | Alcohol misuse, drug misuse, sexual risk behaviour, smoking | Socio-economic status | Clustering |
| McAloney *et al.* (2010)  Northern Ireland | Young adults (N=1,132) | Prospective Cohort | | | Belfast Youth Development Study | Drug misuse, sexual risk behaviour, smoking | - | Clustering |
| McAloney (2015)  Northern Ireland | Young adults (N=875) | Cross-sectional | | | Northern Ireland Life and Times Survey | Drug misuse, alcohol misuse, smoking | Gender | Clustering |
| Parkes *et al.* (2007)  Scotland | Young adults (N=1,322) | Cross-sectional | | | 1996-1999 | Alcohol misuse, drug misuse, sexual risk behaviour, smoking | - | Clustering |
| Sutherland & Willner (1998)  England | Young adults (N=540) | Cross-sectional | | | 1997 | Alcohol misuse, drug misuse, smoking | - | Co-occurrence |
| **Student populations (study inclusion criteria restricted to undergraduate students)** | | | | | | | | |
| Balabanis (2002)  England | Students (N=196) | | Cross-sectional | | Not reported | Alcohol misuse, gambling, smoking | - | Clustering |
| Dodd *et al.* (2010)  England | Students (N=410) | | Cross-sectional | | 2008 | Alcohol misuse, low level of physical activity/exercise, low level of fruit and vegetable intake, smoking | Gender, age, ethnicity | Clustering |
| Underwood *et al.* (2010)  England | Dental Students (N=258) | | Cross-sectional | | 2008 | Drug misuse, smoking | - | Clustering |
| **Older adult populations (study inclusion criteria restricted to ages 50 years and over)** | | | | | | | | |
| Shankar *et al.* (2010)  England  Smith *et al.* (2015)  England | Adults aged ≥50 years (N=11,214)  Adults aged ≥60 years  (N=5,022) | Prospective Cohort | | English Longitudinal Survey of Ageing:  Shankar et al (2010): 2002  Smith et al (2015): 2012 | | Alcohol misuse, low level of physical activity/exercise, smoking (Shankar et al 2010)  Smoking, physical activity (Smith et al 2015) | Education, subjective social status, income (Shankar et al 2010) | Co-occurrence and Clustering |
| **At-risk populations (study inclusion criteria restricted to specific groups at greater risk of engaging in multiple risk behaviours)** | | | | | | | | |
| Cooper *et al.* (2013)  England | Pregnant women in socio-economically deprived city (Bradford)  (N=~12,000) | Cross-sectional | | 2007-2010 | | Smoking and binge-drinking | Ethnicity | - |
| Kelly *et al.* (2014)  Spain | British casual workers in Ibiza, Spain | Cross-sectional | | 2009 | | Smoking, drug misuse, alcohol misuse, sexual risk behaviour | - | Clustering |
| Bolding *et al.* (2006)  England | Men who have sex with men (N=1,307) | Cross-sectional | | 2002-2005 | | Drug misuse, sexual risk behaviour | - | Co-occurrence |
| Melendez Torres *et al.* (2016)  England | Men who have sex with men (N=1,874) | Cross-sectional | | 2011 | | Sexual risk behaviour, drug misuse, alcohol misuse | - | Clustering |
| Fear *et al.* (2007), Thandi *et al* (2015)  UK | UK Armed Forces (N=8,686) | Cross-sectional | | 2004-2006 and 2007-2009 | | Alcohol misuse, smoking | - | Clustering |
| Plant *et al.* (1990)  Scotland | Sex workers (N=205) | Cross-sectional | | 1988-1989 | | Alcohol misuse, sexual risk behaviour | - | Clustering |
| Thomas *et al.* (1990)  Scotland | Clients (mainly male) of sex workers (N=209) | Cross-sectional | | 1988-1989 | | Alcohol misuse, drug misuse, sexual risk behaviour, smoking | - | Co-occurrence |
| Singleton *et al.* (2003)  England, Wales | Prisoners (N=3,563) | Cross-sectional | | Psychiatric Morbidity among Prisoners Survey 1998 | | Alcohol misuse, drug misuse, smoking | - | Co-occurrence |
