# Supplementary material for: A systematic review on the clustering and co-occurrence of multiple risk behaviours
Source: BMC Public Health. 2016 Jul 29;16:657. doi: 10.1186/s12889-016-3373-6 (PMC4966774; doi:10.1186/s12889-016-3373-6)
Supplement: Additional file 3: — Summary of quality assessments for each individual study. (DOCX 26 kb) [file 12889_2016_3373_MOESM3_ESM.docx]

**Supplementary file 3: Summary of quality assessments for each individual study**

| **Study author (year of publication)** | **Appropriate study population** | **Appropriate outcomes** | **Clear aims** | **Appropriate study method** | **Representative sample** | **All explanations of effects considered** | **Good response rate** | **Rigorous development of questions** | **Appropriate choice and use of statistical methods** | **Applicability to national situation** | **Applicability to specific UK region** | **All important outcomes/results considered** |
| --- | --- | --- | --- | --- | --- | --- | --- | --- | --- | --- | --- | --- |
| Aicken (2011) | yes | yes | yes | yes | yes | yes | yes |  | yes | yes | not applicable | yes |
| Balabanis (2002) | yes | yes | yes | can't tell | can't tell | can't tell | can't tell  Not reported | yes | yes | no | no | no |
| Bolding (2006) | yes | yes | yes | yes *Although we do not know whether questionnaire administrators and/or participants were blinded, or whether differences in location (e.g., at home vs clinic/gym) may have influenced questionnaire responses?* | no *The study population were homosexual males, recruited at gyms or HIV testing clinics - not all homosexual men attend these places.* | no *Perhaps additional variables (e.g., peer influence, partner influence, finances, etc) may have influenced the results.* | yes *Sub-sample response rates ranged from 50% to 72% (across the years they were recruited).* | can't tell *There was no description of the questionnaire's development.* | no *They did not adjust for all potentially confounding variables in their analyses.* | can't tell | can't tell | no *They only looked at unprotected anal intercourse with partners - there are several other sexual risk behaviours that are also common - e.g., number of sexual partners.* |
| Buck (2012) | yes | yes | yes | yes | yes | yes | can’t tell | yes | yes | yes | not applicable | yes |
| Cooper (2013) | Yes | yes | yes | yes | can’t tell  Its one multi-ethnic city (Bradford) unclear how representative of multi-ethnic cities in UK | no  Only looked at ethnicity as a factor associated with risk behaviours | can’t tell  Not reported | can’t tell  Not reported | no  Could also have assessed clustering. And also could have adjusted for other factors including socio-economic status | yes | yes | No  Could have assessed other predictors of risk behaviours. Also could have assessed clustering of risk behaviours. |
| Dodd (2010) | yes | Yes | yes | yes | no *The study population was a convenience sample, recruited at a UK university.* | can't tell *They did chi-squared and ANOVA tests to see differences in cluster membership according to ethnicity, gender and age. However, religion may have influenced cluster membership?* | can't tell *Not reported.* | yes | yes *Although analyses could have been adjusted for other factors.* | no *The results are specific to students within one UK university.* | no *The results are specific to students within one UK university.* | yes |
| Egginton (2002) | yes | yes | yes | yes | can't tell | no *Variables like social class, ethnicity, religion, etc, were not controlled for.* | can't tell | yes | no | no | can't tell | yes |
| Fear (2007) | yes | yes | yes | yes | yes *Seems to be. Included a random representative population from the UK armed forces.* | yes *Adjusted for age, rank, service, deployment status, role within parental unit, marital status, children living at home, educational qualifications, smoking status amd having a parent with a drink or drug problem.* | yes *60%* | yes | yes | no | no *The relevant analyses were specific to UK armed forces personnel only. This was also a national sample and was not specific to one UK region.* | yes |
| Griffiths (2010, 2011) | yes *the whole Britain* | yes | yes | yes | yes | can't tell | yes | yes *same survey had been used in 1999.* | can't tell | yes | not applicable | no *They could have included smoking as one of the predictors in the regression model.* |
| Hale (2013) | yes | yes | yes | yes | yes  Longitudinal Study of Young People in England | can’t tell  Limited information on methods | can’t tell  Not reported | yes | can’t tell  Not reported | yes | yes | can’t tell |
| Jackson (2012) | yes | yes | yes | yes | can’t tell | yes | yes | can’t tell | yes | can’t tell | can’t tell | yes |
| Kelly (2014) | yes  Although unclear how big the population of British casual workers in Spain is | yes | yes | yes | no  Opportunistic sample | No  Didn’t assess factors predicting risk behaviours | Yes  82% | can’t tell | no  Could have adjusted for further factors | can’t tell | can’t tell | no  Other variables could also have been taken into account |
| Lawder (2010) | yes | yes | yes | yes | yes | no *Although they performed adjusted analyses for examining predictors of clusters, they did not perform adjusted analyses for analyses examining the clustering of risk behaviours.* | yes | yes | yes *Only for analyses examining predictors of risk behaviour clusters.*  no *In relation to the unadjusted analyses for clustering of risk behaviours.* | can't tell *The study sample was Scottish, not sure how comparable Scottish and other UK populations are? Perhaps these results would also be relevant to English/Welsh/Northern Irish populations?* | yes *Data were collected in Scotland, via a multi-stage stratified probability sampling method.* | yes |
| Liao (1995) | yes | yes | yes | yes | no *Specific to 45-year old women in South London only.* | no *They don't seem to have adjusted for socio-demographic variables such as occupational social class, employment status, ethnicity, marital status, etc. However, this was probably not possible given the nature of the analyses conducted (Chi-squared).* | yes | no *Some, but not all of the questionnaire items had been previously validated.* | no | no | can't tell | can't tell *The results aren't presented clearly per relationship examined.* |
| McAloney (2010) | yes | yes | yes | can't tell | can't tell | no | can't tell | can't tell | no | can't tell | can't tell | yes |
| McAloney (2015) | yes | yes  But measured sexual activity rather than sexual risk behaviour so this outcome wasn’t included in our review | yes | yes | yes | can’t tell  Could have adjusted for factors other than gender | can’t tell  Not reported | yes | yes | yes | yes | yes |
| Melendez-Torres (2016) | yes | yes | yes | yes | can’t tell | no  Could have adjusted for other factors | can’t tell | can’t tell | yes  Although could have adjusted for other factors | yes | yes | yes |
| Parkes (2007) | yes *pupil in Scotland 14-16 yrs* | yes | yes | yes | can't tell | yes *adjusted for gender and social background* | can't tell | can’t tell | no | can't tell | can't tell | yes |
| Plant (1990) | yes | no *The outcomes are not very well defined.* | yes | yes | no *It may be representative of sex workers in Edinburgh, however it would not be representative elsewhere.* | no *They don't seem to have adjusted for any confounding variables such as age, gender, etc. The description of the results and analysis is very vague.* | can't tell *No response rate reported. Not sure if this is applicable given the snowballing technique they used for recruitment, and the nature of the study (face-to-face interviews with sex workers and their clients).* | can't tell *This is possible, they reported that the interview used was a standardised one.* | can't tell *The description is far too vague.* | no | can't tell *Possibly Edinburgh only.* | no *There are no results relating to relationships between the other behaviours investigated, i.e., smoking, illicit drug use.* |
| Plant (2002) | yes | yes | yes | yes | yes | can't tell | can't tell *not reported* | can't tell | can't tell | yes | can't tell | can't tell *The authors gave a very short, concise description of some of their statistical analysis. It was unclear whether they omitted any findings.* |
| Poortinga (2007) | yes | yes | yes | yes | yes *England only.* | yes *(analyses investigating predictors of clustering)*  no *(clustering analyses)* | can't tell *Not reported.* | yes | yes *Although perhaps more confounding variables could have been controlled for?* | yes *England only.* | yes *All English regions.* | yes |
| Randell (2015) | yes | yes | yes | yes  Although could have further explored clustering | can’t tell | yes | can’t tell | can’t tell | yes  Although could have further explored clustering | can’t tell | yes | yes |
| Sabia (2009) | yes | Can’t tell  *The alcohol consumption variable was confusing.* | yes | yes | can’t tell | can’t tell | can’t tell | can’t tell  *Not reported.* | can’t tell | can’t tell | can’t tell | can’t tell |
| Shankar (2010) | yes | yes | yes | yes | yes | yes | can't tell | yes | yes | yes | yes *England* | can't tell |
| Singh (2013) | yes | yes | yes | yes  Although could have further explored clustering | yes | no  Could have examined other predictors | can’t tell | can’t tell | yes | yes | yes | yes |
| Singleton (2003) | yes *prisoners of ENgland and Wales* | yes | no | can't tell | yes | no | yes | can't tell | no | can't tell | not applicable | can't tell |
| Sutherland (1998) | yes | yes *Although the risk thresholds do not include quantities and are therefore a bit vague.* | yes | yes | no *Five schools from such a variety of areas within England cannot be considered representative for any area?* | no | can't tell | can't tell *Not reported.* | no *More sophisticated analytical techniques would have served better for investigating the research questions. The current ones did not allow for adjustment of potentially confounding variables.* | can't tell | can't tell | yes *It is not clear why they amalgamated cigarette and illicit drug use as one measured variable with alcohol consumption as the other variable.* |
| Tang (1997) | yes | yes | yes | yes | no *Perhaps for Bedfordshire only.* | can't tell | can't tell *not reported* | yes *Standardised protocols were used and the Dietary Instrument for Nutritional Education questionnaire was  administered. Physiological measures included blood pressure, height, weight, and serum lipid levels. It was unclear whether the questions relating to exercise, smoking and alcohol habits had been previously piloted/validated.* | yes *Perhaps more confounding factors could have been controlled for?* | no | yes *Perhaps within Bedfordshire alone.* | no |
| Thomas (1990) | yes | can't tell *Risk thresholds aren't very clearly defined (e.g., levels of condom use).* | yes | yes | no | no *Very little adjustment for confounding factors, e.g., age, income, social class...would this information have been possible to obtain given the nature of the study?* | can't tell | can't tell *Though the authors stated that it had been a standardised interview schedule?* | no | no | no | yes |
| Thompson (1992) | yes | yes | yes | yes | yes *England, Scotland and Wales only.* | no *None of the relevant analyses were adjusted for counfounding factors.* | can't tell *not reported.* | can't tell *not reported* | no *Lack of adjustment for confounding factors in the analyses, though the (basic) statistical methods chosen were appropriate for the study aim.* | can't tell *Very little socio-demographic detail about the sample was provided. Seems to be representative though: e.g., "The respondents were chosen at random from representative constituencies in England, Scotland, and Wales.* | can't tell *Very little sociodemographic detail was provided. Difficult to know which regions were sampled - they were not stated.* | yes |
| Thompson (1999) | yes | yes | yes | yes | yes | yes *They did not adjust for social class but this could have been covered by variables such as occupation, employment and household tenure?* | yes | can't tell *The survey instrument was developed by the Health Education Authority - no other details were provided.* | yes | yes *England only.* | not applicable | yes |
| Thornton (1994)  Thompson (1992)* | yes | yes | yes | yes | yes | can't tell *not sure* | can't tell *not reported.* | can't tell *not reported.* | can't tell | yes | not applicable | yes |
| Uitenbroek (1993) | yes | yes | yes | yes | can't tell | no *only age and occupation* | yes |  | can't tell | can't tell | can't tell | yes |
| Uitenbroek (1994) | yes *18 to 51* | yes | yes | yes | can't tell | no | yes *i thinks its ok 67.5% London and 75.2% Scotland* | can't tell | no | can't tell | can't tell | yes |
| Underwood (2007) | yes | can't tell | yes | yes | yes *vocational dental practitioners* | no | yes | yes *piloted in 2000* | can't tell | no *only dental practitioners* | not applicable | can't tell |
| Underwood (2010) | yes | no | yes | yes *In the lecture theatre, participants were spaced apart from their peers in order to ensure confidentiality for responses.* | no *Was specific to one English university only.* | no *No confounding factors (e.g., social class, ethnicity, age, gender) were adjusted for in the analysis of interest.* | yes | can't tell *Details of the questionnaire's development were not reported.* | no *They did not adjust for any confounding variables. The description of the statistical analysis is extremely vague (pretty much non-existent).* | no | no | can't tell *Difficult to say because we don't know much about their statistical analysis techniques?* |
| Wadsworth (2004) | yes | yes | yes | yes | can't tell |  | no | can't tell *The questionnaire was based on another questionnaire - difficult to know whether the questions had been previously validated/piloted.* | yes *see linked study* | can't tell *Data are from two cities in Wales.* | can't tell *Data are from two cities in Wales.* | yes |
| Woodward (1994) | yes | no *Not many of the variables tested actually have risk thresholds - the definitions are very vague.* | yes | yes | yes | no *They did adjust for age, sex and occupational social class - however variables such as education, ethnicity and religion might also influence such results.* | yes | can't tell *Some appeared to have been developed from standardised measures (e.g., the food frequency questionnaire) but others seemed to have been developed for the questionnaire (e.g., the item pertaining to physical activity).* | no | can't tell | yes | no |
